# 87. Cardiac presentation of BehÇet's disease: the impact of HLA-B51 positivity in early diagnosis

**DOI:** 10.1093/rap/rky034.050

**Published:** 2018-09-20

**Authors:** Qainat R Adamjee, Osama M Hamid, Yasmin Bashir, Bernard C Colaco

**Affiliations:** 1 Rheumatology, Central Middlesex Hospital, London, UNITED KINGDOM; 2 Medicine, Imperial College London, London, UNITED KINGDOM

**Introduction:** Behçet's disease (BD) is a rare chronic systemic inflammatory disease associated with vasculitis, and arterial, venous and cardiac disorders. Described in 1937 as a triple-symptom complex of recurrent oral aphthous ulcers, genital ulcers and uveitis (Hulusi Behçet), which remain key diagnostic parameters. Disease prevalence in the UK is rare, with approximately 2,000 case presentations, with BD cases observed more amongst populations living along or originating from the historic silk road road which extends from eastern Asia to the Mediterranean basin. Cardiac involvement, comprising of endocarditis, myocarditis, myocardial infarction and valvular disease are rare (7-46% of BD cases), with pericarditis being the most common, and is usually seen in patients between the ages of 20-40 years. This case series describes an atypical presentation of cardiac manifestations in two patients that resulted in a late diagnosis of BD and the impact of HLA-B51 in diagnosis.

**Case description:** Patient 1: A 46 year old male of Arab ethnicity, had recorded a recurrence of mouth ulcers in 2002, and in 2012 he was diagnosed with viral pericarditis, and subsequently he developed constrictive pericarditis and heart failure in July 2016. Consequently he was tested for the HLA B51/B52 genetic marker, with positive result and was clinically diagnosed with BD. He was prescribed oral prednisolone 20mg od in August 2016 , colchicine in July 2016 and bumetanide 0.5 mg od in September 2016. Previous sensitivities to azathioprine, and frusemide ITP. He was referred to rheumatology in October 2016, and presented with weak paradoxes on inspiration and reduced jugular venous pressure (JVP), with resting heart rate 78. He was commenced on methotrexate 5mg once weekly to increase to 10mg once weekly and bi-weekly blood monitoring agreed. In a four-week follow up he was found to be tolerating methotrexate well with a minor sensation of fatigue. Bloods were stable (reduced 84 ESR, 4mm CRP 2, slight increase in platelets 146). His chest pains continued to improve, with reduced paradoxes and a cardiac MRI was planned for January 2017. In December 2016 he was reviewed by cardiology following results of his echocardiogram. Results showed less pronounced haemodynamic effects. Nothing of clinical concern, and another echocardiogram and CMR requested for follow up in January 2017. Due to renal abnormalities in blood results, bumetanide was stopped in December 2016. Right and left heart catheters were planned in January 2017, and cardiac monitoring continued. Due to reduced thickness of the shaggy pericardium, a pericardiectomy was recommended in February 2017, which he later agreed to despite concerns over the impact it would have on the new job he was starting.

Patient 2: A 27-year-old South Asian female patient, following a period of viral flu, developed chest pain and became short of breath in February 2014. A blood test showed troponins of 400, and she was diagnosed with viral myocarditis following an MRI scan. Initial treatment was with ibuprofen and perindopril. A myocardial biopsy in October 2014 excluded viral aetiology, but showed mild fibrotic change. Following a relapse in February 2014, she was treated with colchicine OD which was effective and the patient reported no chest pain for several months, until it was discontinued in August 2014. She was prescribed oral prednisolone in January 2016. She was referred to rheumatology in August 2016, and it was decided that steroids would be weaned off since she was showing side effects such as weight gain, mooning of the facies and thinning hair. It was decided that colchicine would replace the steroids, and on commencing this, the patient immediately reported that the severity of her chest pain had dropped to 1/10, an improvement that was better than at any point than when she was on steroids. Her troponin level also dropped down to 30. As well as the improvement in response to colchicine, on examination it was found that she had a dry mouth and tongue. She only had occasional mouth ulcers unrelated to the myocarditis flares and no peripheral arthralgia or myalgia. In a genetic test, she was also found positive for the HLA-B51 serotype. Despite the atypical presentation, the unexplained myocarditis, good response to colchicine and HLA-B51 positivity suggested a diagnosis of BD. Gout was excluded on the basis of a uric acid reading of 207 umol/L. In terms of family history, her parents are second cousins while her maternal grandparents are first cousins. She has a family history of HOCM - two sisters out of three siblings have been diagnosed with this.

**Discussion:** Despite cardiac involvement being rare, it has been found that in the months or years following cardiovascular involvement in BD, mortality can be as high as 20%. The diagnosis of BD is challenging since there is no pathognomonic test. However, several criteria to diagnose BD have been proposed. The first widely used set of guidelines used were proposed by the International Study Group (ISG) for BD in 1990. These state that BD requires oral mucosa ulceration, as well as at least two of genital mucosa ulceration, ocular lesion, skin lesion and positive pathergy test. In 2006 the International Criteria for BD (ICBD) was created to provide more updated guidance than the International Study Group (ISG) guidelines. The key difference between the ICBD and the ISG criteria is that the ICBD have incorporated vascular manifestations of BD into the criteria, such as deep vein thrombosis, arterial thrombosis and aneurysm; and ISG criteria has no mention of genetic markers despite HLA-B51 being present in over two thirds of BD patients. However it must be noted that despite the updated guidelines, both the mentioned still lack the inclusion of cardiological factors such as the myocarditis that was present in patient 2 of this case report.

One aspect that supports the theory that BD was the primary cause of resultant cardiovascular diseases in both patients was the use of colchicine. Both patient’s had almost instantaneous relief of chest pain from taking colchicine where more common CV drugs had failed points to an inflammatory cause. Although most commonly used for the treatment of gout, it has also been found to be effective in the treatment of BD. Colchicine works by inhibiting the increased mobility displayed by neutrophils in BD, and therefore has an anti-inflammatory effect. Both patients only had occasional mouth ulcers and no other common features, so would not fit the requirements for BD under ISG criteria. Only with the inclusion of HLA-B51, under ICBD criteria both patients were diagnosed with BD. This lack of inclusion of genetic marker could be a reason for why mean delay in diagnosis was 6 years, and suffered respectively myocarditis and heart failure before the original cause could be identified. It must be further noted it has been found that patients positive for HLA-B51 have their risk of developing BD increased by a factor of 5.90. Despite this case not fulfilling the common criteria required for a diagnosis of BD, a diagnosis could be made on the basis of unexplained chest pain, response to colchicine and HLA-B51 positivity.

**Key Learning Points:** BD is a disease with multi organ involvement and is difficult to diagnose, as several disease manifestations may occur in one patient. The diagnostic use of HLA-B51 in association with Behçet's disease has been clearly indentified, and earlier screening of HLA-B51 may improve clinical outcomes. We recommend that physicians pay close attention to even minor complaints (e.g. unexplained chest pain)and that physical changes be scrutinized and that regular screening protocols are established for the early detection of cardiovascular complications in Behçet's disease, particularly in young patients from this countries where BD is prevalent. This case series shows that BD is a challenging diagnosis to make, and so should be kept in mind when considering differential diagnoses in the case of unexplained myocarditis/pericarditis, particularly in young patients. This is especially important considering that cardiac involvement increases the mortality associated with BD.

**Disclosure: Q.R. Adamjee:** None. **O.M. Hamid:** None. **Y. Bashir:** None. **B.C. Colaco:** None.

